# The Effect of Systematic Clinical Interventions with Cigarette Smokers on Quit Status and the Rates of Smoking-Related Primary Care Office Visits

**DOI:** 10.1371/journal.pone.0041649

**Published:** 2012-07-24

**Authors:** Thomas G. Land, Nancy A. Rigotti, Douglas E. Levy, Thad Schilling, Donna Warner, Wenjun Li

**Affiliations:** 1 Massachusetts Department of Public Health, Boston, Massachusetts, United States of America; 2 Tobacco Research and Treatment Center, Department of Medicine, and Mongan Institute for Health Policy, Massachusetts General Hospital and Harvard Medical School, Boston, Massachusetts, United States of America; 3 Atrius Health/Harvard Vanguard Medical Associates, Newton, Massachusetts, United States of America; 4 Multi-State Collaborative for Health Systems Change to Reduce Tobacco Use, Georgetown, Massachusetts, United States of America; 5 Division of Preventive and Behavioral Medicine, University of Massachusetts Medical School, Worcester, Massachusetts, United States of America; The University of Auckland, New Zealand

## Abstract

**Background:**

The United States Public Health Service (USPHS) Guideline for Treating Tobacco Use and Dependence includes ten key recommendations regarding the identification and the treatment of tobacco users seen in all health care settings. To our knowledge, the impact of system-wide brief interventions with cigarette smokers on smoking prevalence and health care utilization has not been examined using patient population-based data.

**Methods and Findings:**

Data on clinical interventions with cigarette smokers were examined for primary care office visits of 104,639 patients at 17 Harvard Vanguard Medical Associates (HVMA) sites. An operational definition of “systems change” was developed. It included thresholds for intervention frequency and sustainability. Twelve sites met the criteria. Five did not. Decreases in self-reported smoking prevalence were 40% greater at sites that achieved systems change (13.6% vs. 9.7%, p<.01). On average, the likelihood of quitting increased by 2.6% (p<0.05, 95% CI: 0.1%–4.6%) per occurrence of brief intervention. For patients with a recent history of current smoking whose home site experienced systems change, the likelihood of an office visit for smoking-related diagnoses decreased by 4.3% on an annualized basis after systems change occurred (p<0.05, 95% CI: 0.5%–8.1%). There was no change in the likelihood of an office visit for smoking-related diagnoses following systems change among non-smokers.

**Conclusions:**

The clinical practice data from HVMA suggest that a systems approach can lead to significant reductions in smoking prevalence and the rate of office visits for smoking-related diseases. Most comprehensive tobacco intervention strategies focus on the provider or the tobacco user, but these results argue that health systems should be included as an integral component of a comprehensive tobacco intervention strategy. The HVMA results also give us an indication of the potential health impacts when meaningful use core tobacco measures are widely adopted.

## Introduction

The United States Public Health Service (USPHS) Guideline for Treating Tobacco Use and Dependence includes ten key recommendations regarding the identification and the treatment of tobacco users seen in all health care settings [1]. While there is significant research from randomized clinical trials to support the impact of brief tobacco interventions [2], [3], [4], the USPHS Guideline also states that “it is imperative that new research examine the implication of effective treatments in real-world settings.” With the implementation of the Patient Protection and Affordable Care Act (PPACA) and the rapid adoption of meaningful use (MU) compliant software [5] for electronic health records (EHR), it should be possible to use data collected in these real-world settings to measure the health and economic impact of brief tobacco interventions.

While research clearly shows that systems-level changes can reduce smoking prevalence among enrollees of managed health care plans [6], [7], [8], comparable research has yet to emerge from the healthcare delivery system. To date and to our knowledge, there is little quantitative evidence from these real world settings to support the link between the systematic delivery of brief tobacco interventions, behavioral changes, and subsequent health improvements. However, with the volume of data available from electronic health records, we hypothesized that there would be sufficient data to demonstrate that routine clinical interventions with smokers would result in decreases in smoking prevalence and reductions in office visits for smoking related diagnoses.

## Methods

### Setting

This study took place at Harvard Vanguard Medical Associates, a large health care provider network based in eastern Massachusetts. HVMA has more than 20 offices primarily in Boston and the surrounding suburban areas providing primary and specialty health care to more than 400,000 patients. In 2007, HVMA leadership established a clinical quality goal “to intervene” with patients who smoke. A multidisciplinary design team comprised of clinical and administrative personnel, defined “intervention” as identification of cigarette smokers at every office visit and delivery of a brief intervention to each identified smoker during that office visit. HVMA used a team approach to complete the equivalent of the PHS Guideline recommended “5A” tobacco intervention (Ask, Advise, Assess, Assist, and Arrange). There are many international correlates to the 5A model. For example, the National Health Service Stop Smoking program in the United Kingdom recommends a 4A model (i.e., Ask, Advise, Assist, and Arrange follow up). In New Zealand, the system is titled ABC which stands for Ask, Brief advice, and Cessation support. What all these models have in common is that the recommended physician interventions are brief (<10 minutes) and that they include offers of counseling as well as prescriptions for tobacco cessation medications.

**Figure 1:**
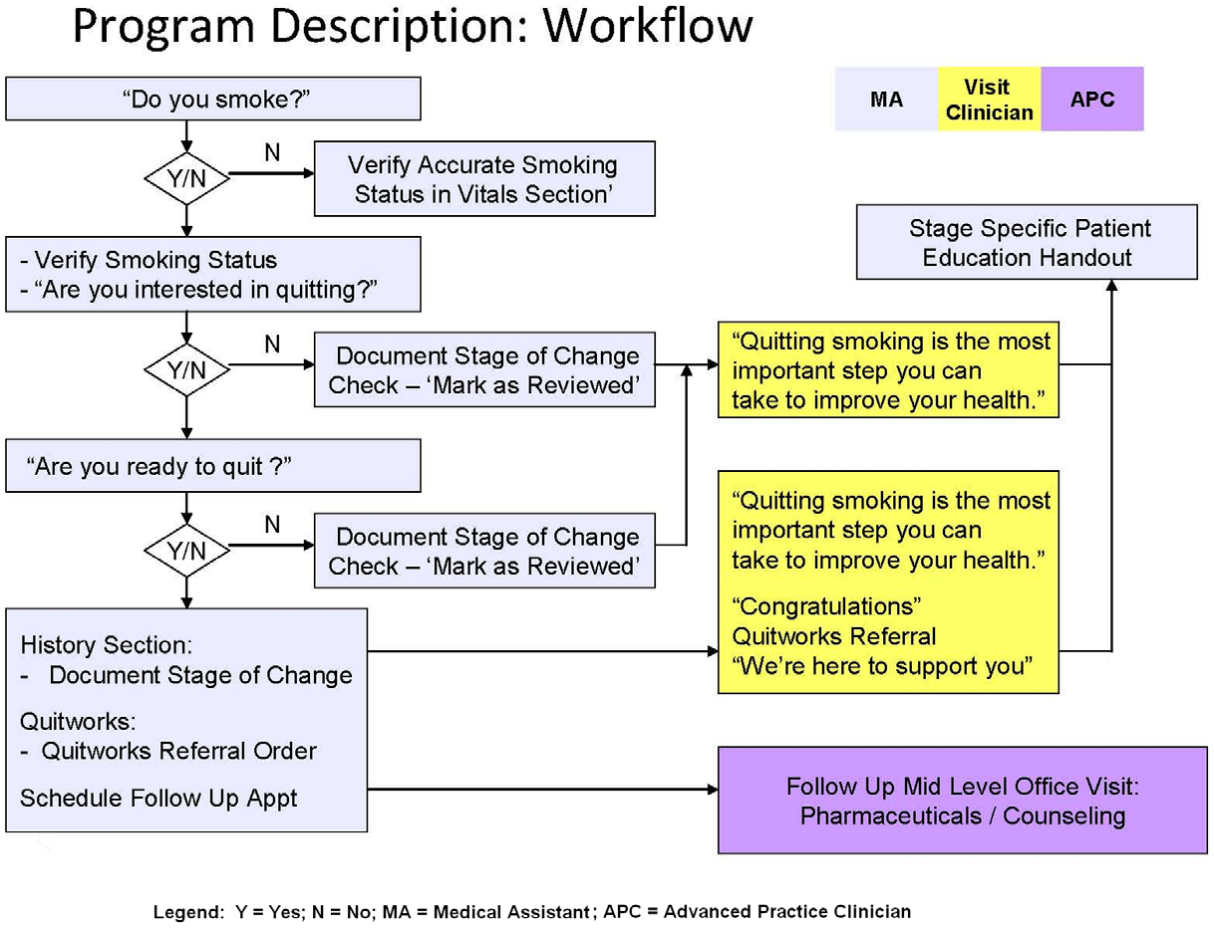
Harvard Vanguard Medical Associates work flow for interventions with smokers.

The data recorded at HVMA focused exclusively on cigarette smokers instead of the broader definition of tobacco users. See Figure 1 for the HVMA work flow for identifying cigarette smokers.

In this work flow, the medical assistant was charged with recording in the EHR smoking status during each office visit and to assess readiness to quit. The clinician was responsible for advising each smoker to stop and for assisting each smoker according to his/her stage of change. Decision support tools for clinicians were embedded into the EHR to promote use of evidence-based medications to strengthen quit attempts. An option to refer smokers to a community-based, state-funded “stop-smoking service” that provides free telephone counseling could be ordered through the EHR. Advance Practice Clinicians (Nurse Practitioners and Physician Assistants) were trained to provide counseling and were educated on the use of stop-smoking medications. Many intervention sites identified a tobacco champion to lead the work locally. Feedback reports of medical assistant performance were delivered to clinical staff and administrative supervisors monthly.

### Data

De-identified encounter level data for all primary care office visits for all adult patients at 17 HVMA sites was prepared by analysts from Harvard Vanguard Medical Associates (HVMA). Records covered the period from 1/1/2005 through 11/30/2010. Evaluation plans were reviewed and approved by the Institutional Review Board for the Massachusetts Department of Public Health. Harvard Vanguard obtains written consent from patients for the type of analysis conducted here. The consent form includes the following 2 statements.

1. Harvard Vanguard may use or disclose your Health Information in order to conduct its business of providing health care. These “health care operations” may include quality assessment, training of medical students, credentialing and various other activities that are necessary to run our practice and to improve the quality and cost effectiveness of the care that we deliver to you. Some of these activities occur in conjunction and cooperation with other Atrius Health groups. Other of these business operations may be performed by outside parties (“Business Associates”) on Harvard Vanguard’s behalf. Our Business Associates must agree to maintain the confidentiality of your Health Information.
2. Harvard Vanguard may disclose your Health Information for public health activities.

The legal Department at Harvard Vanguard carefully reviewed this project and determined that the analysis fell within the realm of public health work related to quality improvement.

The de-identified data set prepared by HVMA analysts contained demographics and encounter level data for 310,577 adult patients. Demographics for each patient included a randomly defined patient ID, age, race/ethnicity, marital status, town of residence, and patient’s “home” clinic site. All HVMA patients have a “home site” which is the location of their primary care provider. Since patients can change doctors and/or move from one home site to another, the “home” for this data set was the clinic site associated with the patient’s primary care provider on 11/30/2010. Data for all office visits at 17 HVMA office sites between 1/3/2005 and 11/30/2010 also were prepared. Included in the office visit encounter data were the unique patient ID, a primary and four secondary diagnoses, and recorded components of the brief intervention with cigarette smokers. For the 310,577 unique patients, there were 2,561,782 unique single-day patient encounters in the data set prepared by HVMA analysts. Nearly all (96.6%) interventions with cigarette smokers occurred at a patient’s home site.

To be included in our analysis, patients had to be Massachusetts residents between the ages of 22 and 64 on 11/30/2010, who were screened for smoking status at least once. At least 3 years had to elapse between the first and last office visit. The requirement that patients be at least 22 years of age by 11/30/2010 was to ensure that all patients in our data set were at least 18 when the first HVMA site began to systematically intervene with cigarette smokers (1/1/2007). Although some children were screened for smoking status by HVMA, the intervention program for cigarette smokers was aimed almost exclusively at adults. The upper age limit was set to 64 because it was thought that older patients might be receiving a greater proportion of their care outside the primary care system. Seasonal residents and college students might also receive care outside the primary care system, but it was impossible to screen only for full-time/non-college residents with the data available.

In the original data set, 104,639 of the 310,377 patients met the criteria described above. Of this total, 15,286 had some history of self-reported smoking recorded in the EHR between 1/3/2005 and 11/30/2010 while 89,353 had no recorded history of current smoking in that time.

When recorded, smoking status was listed as either “Yes”, “Quit”, “Never”, “Passive” or “Not Asked.” Since the “Passive” and “Not Asked” categorizations could not be used to specifically define a patient’s use of cigarettes, these categories were ignored. Therefore, smoker identification for this study was defined as an office visit where smoking status was listed as “Yes”, “Quit”, or “Never.”

The recorded smoking status was attached to the dated office visit. This date was not overwritten as is often the case when smoking status is stored in a patient’s social history. As a result, the full complement of patient encounter records would have a discontinuous but longitudinal history of smoking status. In addition to smoking status, information could be recorded about a patient’s interest in quitting, readiness to quit, smoking pattern, referrals to telephone counseling, and prescriptions for medications covered by insurance.

For this study, a brief intervention with smokers was defined as any evidence at a specific visit that the conversation about cigarette smoking went beyond the identification of smoking status. This could include information about interest in quitting, readiness to quit, smoking pattern, referrals to telephone counseling, and prescriptions for medications covered by insurance. Therefore, any visit in which a patient was identified as a smoker could also include a brief intervention about smoking.

### Assessing Data Quality

To assess data quality, we focused on all office visits throughout the data set where a patient’s smoking status was recorded as “Never.” Of the three primary categories of smoking status (i.e., “Yes”, “Quit”, and “Never”), “Never” is the only absolute classification. Logically, no visit where status is recorded as “Never” should have any other status recorded at a prior visit. To obtain our quality assessment score, we counted the number of times that the smoking status was also recorded as “Never” where smoking status was also recorded at an earlier visit. Next, we computed the percentage of the earlier visits in which smoking status was also recorded as “Never.” For the 384,338 visits at which smoking status was recorded as “Never”, 381,917 (99.4%) also had smoking status recorded as “Never” at the earlier visit.

### Establishing the Date for Systems Change and Defining Impact

To our knowledge there is no common standard for defining systems change using real-world office encounter records. Operationally, we defined “health systems change” as first month when more than half of all office visits at a given site included an identification for cigarette smoking. In all months following that date, the rate of cigarette smoker identifications could never drop below 50%. Furthermore, there had to be at least 12 consecutive months with rates above 50%. By this definition, 12 of the 17 HVMA sites had achieved “systems change.”

Changes in self-reported smoking behavior were examined by computing the proportion of all patients who were recorded as smokers at the earliest possible visit and then comparing this to the proportion of all patients recorded as smokers at the latest possible visit. Group comparisons were made between sites that achieved systems change and those that did not.

Changes in the rate of smoking-related office visits were analyzed using generalized estimating equations (GEEs) with a logistic link function and patient as the unit of analysis. The period between 1/3/2005 and 11/30/2010 was divided into 77 twenty-eight day segments for 104,639 patients –15,286 patients with histories of recent smoking and 89,353 patients with no recent history of self-reported smoking. The dependent variable was the presence or absence of a smoking-related office visit during the 28-day period. If any of the first five recorded ICD9 codes for any visit in a 28-day period matched the list of smoking-related diagnoses from the Surgeon General’s 2004 report on Smoking and Health [9], the period was coded as 1. All other periods were coded as 0.

To avoid biasing the results, data prior to and including the period of the first recorded visit were not included in the analysis. Patients were divided into those who had some history of self-reported smoking between 1/5/2005 and 11/30/2010 and those who had no history of self-reported smoking. Any patient who reported current smoking at any visit between 1/5/2005 and 11/30/2010 was grouped with the smokers. Longitudinal data for smokers and non-smokers were evaluated separately.

There were three temporal variables in each model: time since 1/1/2005, time since the first recorded office visit, and time since systems change occurred at the patient’s home site. This last variable was the primary focus of our analysis. We hypothesized that there would be decrease in the rate of smoking-related office visits following systems change and that this effect would only be seen in patients with a recent history of self-reported smoking.

Our model adjusted for the seasonality of office visits using sines and cosines [10]. Since the number of office visits generally increases during flu season, our model also included the percentage of office visits for all patients seen in a specific 28-day period in which a flu vaccine was given as well as the percentage of all office visits in which influenza was among the 5 recorded diagnoses. The model included the cigarette tax rate throughout the period as well as a binary variable for the implementation of health insurance reform in Massachusetts (7/1/2006). There were 2 tax increases between 1/5/2005 and 11/30/2010. To adjust for the individual rate of office visits by patients, the model included a term for the average number of visits for a specific patient in all prior periods. Finally, we adjusted for correlations among repeated office visits within patients across time, assuming a first-order autoregressive structure. All analyses were conducted using SAS 9.1 (SAS Corporation, Cary, North Carolina).

## Results

### Rates of Identification and Brief Intervention Before and after Systems Change

As defined above, “systems change” occurred at 12 of the 17 sites between 1/1/2007 and 4/1/2009. For all 12 sites, there was a dramatic and significant increase in the identification rate of cigarette smokers after the date of achieving systems change. All but one site achieved an 80% identification rate within 9 months of that date. The median time between the date of “systems change” and an 80% identification rate was just 4.5 months. At 11 of the 12 sites, there was also a significant increase in the percentage of rate of brief intervention for identified cigarette smokers. Identification rates for the remaining 5 sites remained relatively low throughout the study period. At sites that achieved systems change, 82.5% of visits where patients were identified as smokers included evidence of a further clinical intervention. At sites that did not achieve systems change, this rate was only 59.4%. Table 1 presents pre-post identification and intervention rates at the 17 HVMA sites.

**Table 1:** Identification and intervention rate by site before and after “systems change”.

		Before Systems Change			After Systems Change		
Site	Date of “Systems Change”	Total Visits	Identification Rate	Brief Intervention Rate	Total Visits	Identification Rate	Brief Intervention Rate
Site 1	Jan-07	20,638	21%	50%	84,438	91%	84%
Site 2	May-07	47,651	3%	57%	77,419	93%	94%
Site 3	Jun-07	64,552	3%	43%	102,194	94%	91%
Site 4	Jul-07	88,015	12%	75%	126,955	88%	84%
Site 5	Oct-07	1,906	31%	47%	2,777	79%	63%
Site 6	Jun-08	69,262	13%	70%	73,054	87%	88%
Site 7	Jul-08	157,212	14%	64%	129,824	93%	89%
Site 8	Aug-08	93,455	9%	57%	65,491	91%	91%
Site 9	Oct-08	62,736	9%	72%	41,214	81%	84%
Site 10	Feb-09	130,338	20%	67%	74,421	92%	92%
Site 11	Mar-09	33,210	18%	47%	19,588	64%	57%
Site 12	Apr-09	76,866	13%	59%	36,504	87%	88%
Site 13	N/A	255,254	34%	61%	N/A	N/A	N/A
Site 14	N/A	134,036	25%	54%	N/A	N/A	N/A
Site 15	N/A	167,814	21%	63%	N/A	N/A	N/A
Site 16	N/A	44,878	15%	68%	N/A	N/A	N/A
Site 17	N/A	280,080	11%	51%	N/A	N/A	N/A

The proportion of home-site interventions was slightly higher for patients seen at the 12 sites where systems change took place (97.2% versus 93.1%).

### Demographics, Smoking Prevalence, and Office Visit Statistics

Most demographics were similar for smokers and non-smokers. However, patients with a recent history of self-reported smoking were more likely to be younger, male, of white race and live alone. Smokers had a significantly higher average number of office visits (Table 2).

**Table 2:** Demographics and office visit statistics by smoking status.

	All Patients(n = 104,639)	Smokers(n = 15,286)	Non-Smokers(n = 89,353)
Criteria*	Smoking status recorded between1/5/05 and 11/30/10.	Evidence of current smokingbetween 1/5/05 and 11/30/10.	No evidence of current smoking between 1/5/05 and 11/30/10.
Average age in years	46.5	45.3	46.8
% Female	61.1%	58.0%	61.6%
% Single	20.0%	25.5%	19.0%
% Married	47.9%	35.3%	50.1%
% White non-Hispanic	68.0%	72.0%	67.3%
% Black non-Hispanic	12.5%	12.4%	12.5%
% Hispanic	4.1%	3.8%	4.2%
Average number of office visits	11.9	13.3	11.7
Average time between first and last visit	4.6 years	4.7 years	4.6 years

* Full inclusion criteria required that patients be Massachusetts residents between the age of 22 and 64 on 11/30/2010, screened for smoking status at least once, with at least 3 years between the first and last office visit.

Changes in smoking prevalence were examined by focusing on patient visits where smoking status was recorded. Of the 104,639 patients in our study group, 13,517 (12.9%) were current smokers at the first visit where smoking status was recorded. On the last visit where smoking status was recorded, 11,817 (11.3%) were current smokers. Overall, there were 1,700 (12.6%) fewer smokers at the last visit. The decrease in self-reported smoking prevalence was 40% larger at the 12 sites that achieved systems change (13.6% vs. 9.7%, p<.01). As one would expect given our operational definition of systems change, patients received more clinical interventions about cigarette smoking at sites that achieved systems change (5.3 vs. 2.6, p<0.001).

The impact per encounter of brief clinical intervention on the likelihood of quitting was examined using a logistic model. The outcome variable was the final recorded smoking status for a patient (i.e., Yes = 1, Quit = 0). The model included as predictors the total number of office visits where the patient’s smoking status was recorded and the number of office visits where smoking status was not recorded. The analysis was restricted to 1,255 patients who had at least 4 years of between the first and last visit, at least 3 years between the first and last confirmation, and at least one visit in which smoking status was recorded as “Yes.” Each encounter where a smoker’s smoking status was recorded increased the likelihood of quitting by 2.6% (p<0.05, 95% CI: 0.1%–4.6%).

### Decreasing Likelihood of Smoking-Related Office Visits for Smokers

Most office visits did not include a smoking-related diagnosis code. On average, smokers had 0.90 smoking related office visits throughout the time period studied while non-smokers had an average of 0.69 visits with a smoking-related diagnosis code. Changes in the rate of smoking-related office visits were computed using generalized estimating equations (GEEs). Separate models were developed for patients with recent histories of self-reported smoking and all other patients. We refer to these groups as smokers and non-smokers. The independent variable of primary interest was the time since systems change occurred at the patient’s home site.

After adjusting for temporal effects, seasonality, previous visit pattern, flu-related visits, the date of health reform, and cigarette taxes, the annualized rate of smoking-related office visits following systems change for smokers decreased by 4.3% (95% CI: 0.5%–8.1%). The difference from the unadjusted to the adjusted rates is likely due to the fact that several of the independent variables had strong positive relationships with the dependent variables (e.g., age, gender, average number of office visits, and flu-related visits). For non-smokers, there was a non-significant decrease (0.8%, p = 0.37) in the annualized rate of smoking-related office visits following systems change (95% CI: −0.4% to 2.0%). See Table 3.

**Table 3:** GEE parameter estimates of percent change for smoking-related office visit in period: smoker and non-smoker models.

	Smokers *(n = 15,286)	Non-Smokers **(n = 89,353)
Variable	Percent change and 95% CI	
Temporal effect	21.8% (14.8% to 28.9%)	22.9% (19.8% to 25.6%)
Years since 1^st^ visit	10.9% (5.6% to 16.2%)	9.9% (8.3% to 11.5%)
Years since systems change	−4.3% (−8.1% to −0.5%)	−0.8% (−2.3 to 0.7%)
Before/After Health Reform	11.7% (−2.7% to 7.5%)	6.4% (−1.2% to 14.1%)
Seasonality 1 (sine)	0.2% (0.1% to 0.3%)	0.2% (0.1% to 0.3%)
Seasonality 1 (cosine)	−0.3% (−0.4% to −0.2%)	−0.3% (−0.4% to −0.2%)
Avg # visits in prior periods	1.5% (1.4% to 1.6%)	1.3% (1.27% to 1.33%)
Current Age	3.0% (2.7% to 3.3%)	2.0% (1.9% to 2.1%)
% of visits in period for flu vaccination	0.3% (0.27% to 0.38%)	0.4% (0.40% to 0.46%)
% of visits in period with flu diagnosis	0.1% (0.05% to 0.15%)	0.04% (0.02% to 0.06%)
Current per pack tax rate	−0.34% (−.49% to −.20%)	−0.36% (−.44% to −.28%)

* Evidence of current smoking between 1/5/05 and 11/30/10.

** No evidence of current smoking between 1/5/05 and 11/30/10.

To explore whether demographics could explain the reduction in the rate of smoking related office visits, six primary and interaction terms were added to the smoker and non-smoker models. The primary terms were the six demographic variables shown in Table 2 (i.e., Gender, Single, Married, White non-Hispanic, Black non-Hispanic, and Hispanic). The interactions were these demographics in conjunction with the time since systems change occurred at a patient’s home site. Of particular interest were the interaction terms. For both models, the only interaction term reaching significance was the time since systems change and whether a patient was married. Specifically, as time since change increased the rate of decrease in the likelihood that a married patient visited the doctor for a smoking related condition increased slightly. The reason for this effect is not clear. That said, the addition of these interaction terms did not appreciably change the main effects seen for systems change in either model. For smokers, there was still a significant decrease in the likelihood of a smoking related office visit following systems change at the home site. For non-smokers, there was no effect.

## Discussion

### Summary of Results

An operational definition of systems change was established for clinical interventions with cigarette smokers. This definition included thresholds for frequency and sustainability. Based on this definition, 12 of 17 HVMA sites achieved systems change between 1/1/2007 and 12/1/2009. These 12 sites had significant increases in rates of smoker identifications and further clinical interventions with smokers. Decreases in smoking prevalence were found across all sites; however, the reduction in prevalence was 40% greater at sites achieving systems change. We estimate that each clinical intervention with a smoker increased the likelihood of quitting by 2.4%. The likelihood of an office visit for a smoking-related diagnosis also decreased but only for smokers at sites that achieved systems change (4.3%). Among non-smokers, there was no significant change in the rate of office visits for smoking-related office visits following systems change. Patient demographics did not appear to strongly affect the likelihood of a smoking related office visit following systems change.

### Implications

The health care system should be viewed as central to the any tobacco intervention strategy. As recommended in the USPH Guideline, health care administrators like practice managers and chief medical officers, as much as individual clinicians, must be responsible for ensuring that tobacco interventions become an integrated component of health care delivery. Yet, despite the well-known consequences of tobacco use and conclusive research on the effectiveness of tobacco treatment, many healthcare facilities still lack the policies and clinical systems needed to achieve consistent and effective treatment. However, this landscape is changing rapidly. Recent federal legislation, including PPACA, ARRA (American Recovery and Reinvestment), and HITECH (Health Information Technology for Economic and Clinical Health) include provisions that incentivize physician providers and hospitals increasingly to identify tobacco users, assess use, and conduct interventions [11], [12]. Chief among them are CMS incentives to achieve the meaningful use of electronic health records which includes identification of smokers as a core measure.

The HVMA data support and shine the spotlight on strategies in healthcare that focus on the system, rather than the individual clinician. In the case with HVMA, data captured in the EHR is retrieved and reported back monthly. Administrators and clinicians (including physicians, nurse practitioners, physician assistant, and medical assistants) are informed about their own performance with comparisons to other sites. This analysis also demonstrates that data sharing with clinicians may go beyond the rate of brief tobacco interventions and enter the realm of behavioral change and improvements of patient population health.

In contrast to strategies that target only the clinician or the tobacco user, with systems strategies, tobacco use interventions are likely to become a fully integrated and routine part of patient care. Bolstered increasingly by meaningful use of EHRs, they may become easier to perform than not. If results such as those realized within HVMA can be replicated across the primary care delivery system, significant strides can be made towards reducing tobacco use prevalence and improving health.

### Limitations

A number of limitations should be noted. Although we endeavored to assess data quality, no independent measure of quality was available and thus the inaccuracy of the electronic medical records may lead to variability as well as potential bias in the analysis. The size of this potential issue cannot be known. However, our test of internal consistency showed that patients who were recorded as never having smoked were also listed as never smokers 99.7% of the time at prior visits. Furthermore, the sites that had increased rates of brief intervention also had simultaneous decreases in the number of smokers and the likelihood of office visits for smokers. This is consistent with literature showing relationships between likelihood of quitting and tobacco use interventions with medical doctors. Had the data quality been poor, it is unlikely that this relationship would have existed in the HVMA patient histories.

This analysis also relied on patient self-report smoking status that is subject to reporting bias, especially among certain populations like pregnant women. The self-report bias may have affected the estimates of smoking prevalence, but unlikely to have affected the estimates of pre-post changes. The percentage of women with ICD9 diagnosis codes (V22) for pregnancies varied across sites from 2% to 5%. There was no discernible pattern between these percentages and sites that achieved systems change.

Without any measure of continuity of care, we also can’t know whether patients visited non-HVMA providers for any period of time. We attempted to deal with the limitation by requiring that at least 3 years elapse between the first and last visit for all patients in our study group. We have no reason to assume that patients who sought routine care elsewhere and then returned at a later date to receive care at HVMA would bias the results in any way.

Similarly, the likelihood of office visits for smoking-related diagnoses could be impacted by patients seeing non-HVMA providers for their care. Smokers, in particular, have more health problems and may require care of specialists or more ED or hospital visits for smoking-related diagnoses. Furthermore, there is extensive literature on what has been called the “ill-quitter effect” or “quitting while sick” [13]. While these effects are certainly real, we sought to limit their impact on our analysis by requiring that the visit history for all patients be at least 3 years long and by restricting our population to adults under 64. It was thought that this specific group of patients would be more likely to obtain health care through the HVMA primary care system. But the most important argument countering this potential limitation is the fact that reductions in office visits with smoking-related diagnoses were found only for smokers and that these changes were a function of the date established for health systems change. Non-smokers showed no reduction in the likelihood of an office visit for smoking-related diseases following systems change.

### Future Directions

Future research in this area should also examine data sets collected in settings other than the primary care setting. Without a better understanding of changes in hospitalization rates, it would be impossible to claim that there have been significant health improvements or to develop adequate return on investment estimates for brief tobacco intervention in real world settings. Nonetheless, the success of the HVMA program of brief tobacco use interventions demonstrates the value of system-wide adoption of MU core tobacco measures. When systems routinely meet the MU criteria, there will be real opportunities to improve healthcare quality, among them tailored feedback systems to motivate clinicians, new ways to identify and address health disparities, and development of payment systems that tie bonuses to reliable measures of improving population health.

The forces driving healthcare in the United States to adopt a systems approach to tobacco interventions are quite large. These include significant federal legislation (PPACA, ARRA, and HITECH), tobacco related meaningful use rules, and the move toward Accountable Care Organizations, Alternative Quality Contracts, and Value Based Purchasing. With these tailwinds, the rates of tobacco interventions in the United States are likely to increase significantly in the coming years, ultimately leading to substantial savings from the decreased utilization of health care services related to tobacco use.
